# Familiarity affects collective motion in shoals of guppies (*Poecilia reticulata*)

**DOI:** 10.1098/rsos.170312

**Published:** 2017-09-20

**Authors:** Scarlet Davis, Ryan Lukeman, Timothy M. Schaerf, Ashley J. W. Ward

**Affiliations:** 1 School of Life and Environmental Sciences, University of Sydney, Sydney, New South Wales, Australia; 2 Department of Mathematics, Statistics and Computer Science, St. Francis Xavier University, Antigonish, Nova Scotia, Canada; 3 School of Science and Technology, University of New England, Armidale, New South Wales, Australia

**Keywords:** shoaling, schooling, collective behaviour, alignment

## Abstract

The coordinated and synchronized movement of animals in groups often referred to as collective motion emerges through the interactions between individual animals within the group. Factors which affect these interactions have the potential to shape collective movement. One such factor is familiarity, or the tendency to bias behaviour towards individuals as a result of social recognition. We examined the effect of familiarity on the expression of collective motion in small shoals of female guppies (*Poecilia reticulata*). Groups comprising familiar individuals were more strongly polarized than groups of unfamiliar individuals, particularly when in novel surroundings. The ability to form more strongly polarized shoals potentially promotes information transfer and enhances the anti-predator benefits of grouping.

## 1. Introduction

Collective motion describes the cohesive and synchronous movements of groups of animals. It is a taxonomically widespread phenomenon and may be observed in a range of different behavioural contexts, including migration, foraging and predator avoidance [1]. Global patterns manifest during collective motion emerge through repeated, localized interactions between group members [2–6]. Often each individual aligns its behaviour with that of its near neighbours as they move; the ultimate effect is to produce a coherent, ordered group where all individuals travel in the same direction and at similar speeds. This coherence not only increases the efficiency of group movement, but may also increase the speed of information transfer through the group and the anti-predator benefits of grouping by enhancing the confusion effect suffered by predators [7,8].

Although many theoretical models have tended to assume that individuals within the group respond to their near neighbours uniformly [1], this represents an oversimplification for the majority of species. We know that animal species are capable of complex social recognition and that individuals are able to differentiate between conspecifics and tailor their responses accordingly. For example, individuals of many social species behave preferentially to conspecifics that they have previously encountered and which they recognize, a phenomenon known as familiarity [1,9]. This can have consequences for their social behaviour, in particular increasing the likelihood of associating with familiar individuals [10–12]. This can produce effects at the level of the group; for example, shoals of familiar fathead minnows are more cohesive compared with shoals of unfamiliar conspecifics [13]. Little is known, however, about how familiarity with group-mates might affect collective behaviour.

In the context of collective motion, one of the key measures of coordination across the group is polarization, the extent to which animals within the group align with one another. Individuals in highly aligned, polarized groups may be able to communicate more effectively with one another, potentially aiding information transfer and, ultimately, the effectiveness of foraging and predator avoidance [14]. We compared patterns of collective motion in shoals of familiar fish and in shoals of unfamiliar fish, examining how group polarization is influenced by social recognition. In addition, we examined the effect of familiarity on cohesion, measured as the nearest-neighbour distance, and speed. Finally, we tested the patterns of collective motion at two separate time periods, firstly when the fish were newly introduced to the arena, and secondly after 30 min, when the fish had likely had time to habituate to their surroundings, a factor which is known to influence shoaling behaviour [15,16].

## 2. Material and methods

### 2.1. Study animal

The guppy is an important model species in behavioural ecology. It is a shoaling species and exhibits collective motion. In addition, females show strong association preferences for familiar individuals[17–19]. Familiarity in this species builds up over a period of 12 days and each individual can become familiar with up to 40 other individuals [17–19]. Just as familiarity develops over time, it can also decline at a similar rate without reinforcement [20]. Guppies for use in this experiment were collected from a feral population near Darwin, Northern Territory, in September 2015 and airfreighted to Sydney where they were held in 200 l stock tanks at the animal housing facility of the University of Sydney. The fish were fed Wardley tropical fish flakes twice daily until satiated. They were held at a temperature of 26°C with 12 L : 12 D lighting regime. To facilitate subsequent identification of individuals, all fish were marked using elastomer (NorthWest Marine Technologies Inc.) under a mild anaesthetic solution.

### 2.2. Experimental protocol

One week after the fish had arrived in Sydney, we collected 72 female guppies from the stock tanks, and distributed them evenly between six 20 l tanks, so that there were 12 fish in each tank. The body length of females used in the experiment was determined from photographs; the range was from 28 to 38 mm. Each tank was furnished with gravel and a plastic plant and was filtered using an air-driven sponge filter. The feeding, temperature and light regime were as described above. The fish were kept under these conditions for two weeks, to allow familiarity to develop.

Following this, we collected groups of four fish for use in experiments. The fish were either all collected from the same tank (familiar) or collected from four separate tanks (unfamiliar). The groups were constructed using a random number generator to determine the tank the fish would be collected from. Then, random sampling determined the fish used from each tank. The group of four fish was then added to a clear, Perspex cylinder measuring 120 mm in diameter, which in turn was situated within a white acrylic annular arena [21,22]. The internal and external diameters of the annulus were 660 mm and 270 mm, respectively, at the water surface, giving the fish a channel of 195 mm to swim in and it had a water depth of 70 mm. The arena was visually isolated using white plastic sheets and lit using daylight LED strips. After 5 min, the Perspex cylinder was raised and the fish were allowed to swim freely throughout the annulus. The fish were filmed from above using a Canon G1X camera, filming at 25 fps with a resolution of 1080p. The fish remained in the arena for a total of 40 min before being removed and replaced with a new group. In total, we tested six groups of unfamiliar fish and six groups of familiar fish. Each fish was tested once, at most, and no fish were reused between trials.

### 2.3. Data analysis

To analyse the behaviour of the fish, we used two 5 min sections of film. The first section of film was taken from the 5 min period immediately following their release and the second from a period 30 min later, i.e. from minutes 35 to 40 of the trial. These 5 min sections were converted to .avi files using VirtualDub (virtualdub.org) and then tracked using IDTracker software [23], providing a series of (*x*,*y*) coordinates for each fish. From these, we calculated individual-level response variables, the median speed and the mean nearest-neighbour distance of each fish, for each of the two sampling periods. We further calculated our group-level response, mean polarization of the group, based on individual trajectories for each group at each of the two sampling periods. These were analysed using the nlme package in R [24]. We analysed our group-level response variable, mean polarization, using a repeated measures model, with social familiarity (familiar versus unfamiliar) as the between-subjects factor and sampling period (after 5 min versus after 30 min) as the within-subjects factor. We analysed our individual-level response variables, median speed (in mm s^−1^; we used the median speed rather than the mean for each individual, because individual speed distributions tend to be positively skewed) and mean nearest-neighbour distance (in mm) also using repeated measures models. To do this, we included individual ID nested within group as a random factor. We assessed normality by inspecting quantile–quantile plots and also using Shapiro tests. As speed and nearest-neighbour distance were marginally non-normally distributed with a slight positive skew, we transformed the data using a log transformation. We used Levene's test to assess the equality of error variances. As we made multiple comparisons of the data, we applied a Benjamini–Hochberg false discovery rate procedure to guard against the possibility of Type I errors.

In addition, we made a more detailed analysis of the interactions between animals within the familiar and the unfamiliar groups. In particular, we examined the alignment of group members, relative to a focal individual positioned at the origin and travelling parallel to the positive *x*-axis. Details of the calculations used are provided in the electronic supplementary material. Finally, because both speed and proximity to group-mates are likely to affect the extent to which individuals are aligned with one another, we investigated the effects of travelling speed and inter-individual distance on the directional correlations of fish within each group. To do this, we calculated the mutual speed of pairs of fish within each group by taking the average of the two fish's speeds and the distance between the pair. Then for each time step, we calculated the directional correlation between the two fish and plotted this as a function of mutual speed and distance, averaging over all observations at a given (discretized) speed–distance location. The approach used to calculate directional correlation is presented in the electronic supplementary material.

## 3. Results

Data used in the following analyses are presented in the electronic supplementary material.

**Figure 1.**
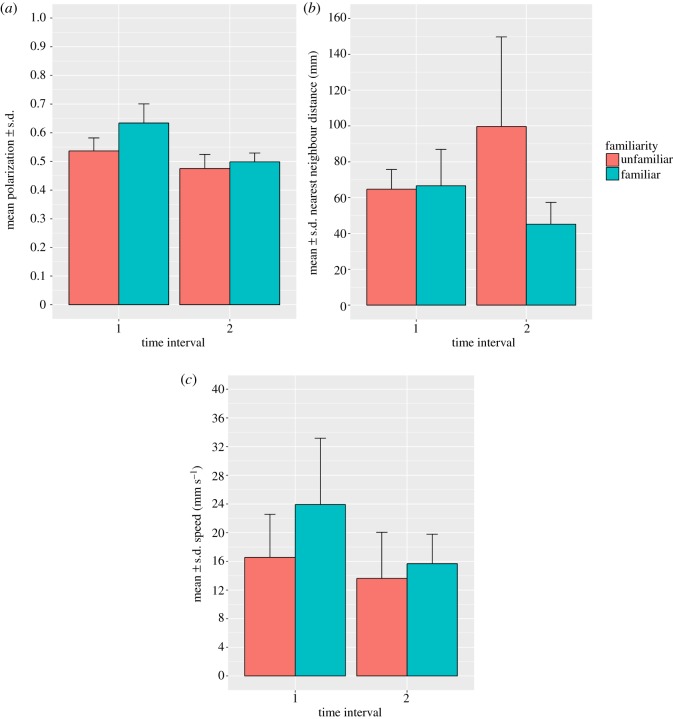
Comparisons of shoals of unfamiliar fish versus shoals of familiar fish at two time intervals (*a*) mean (+s.d.) group polarization (*b*) mean (+s.d.) of the mean nearest-neighbour distances of group members (*c*) mean (+s.d.) of the median speeds of group members.

Groups of familiar fish were more polarized than unfamiliar groups (table 1*a* and figure 1*a*). There was no difference in nearest-neighbour distances, nor was there a significant difference in swimming speed between treatments (table 1*b* and figure 1*b,c*).

**Table 1.** Output of repeated-measures models, showing the effect of social familiarity and sampling period on (*a*) mean polarization and (*b*) median speed and mean nearest-neighbour distance.

			95% CI				
	value	s.e.	lower	upper	*t*	d.f.	*p*-value
(*a*)							
polarization							
familiarity	0.097	0.029	0.034	1.161	3.397	10	0.007^a^
time	−0.061	0.028	−0.124	0.001	2.183	10	0.057
fam × time	−0.074	0.04	−0.164	0.015	1.847	10	0.096
(*b*)							
speed							
familiarity	0.858	0.317	2.144	13.719	2.695	10	0.023
time	−0.828	0.194	−8.653	−2.759	4.268	46	<0.001^a^
fam × time	−0.656	0.274	−11.455	−3.12	0.73	46	0.021
nearest-neighbour distance							
familiarity	−0.123	0.082	−46.569	14.299	1.504	10	0.164
time	0.104	0.048	12.591	47.435	2.17	46	0.035
fam × time	−0.081	0.0677	−48.642	1.059	1.203	46	0.235

^a^Values that are significant at the 0.05 level following application of Benjamini–Hochberg false discovery rate procedure.

**Figure 2.**
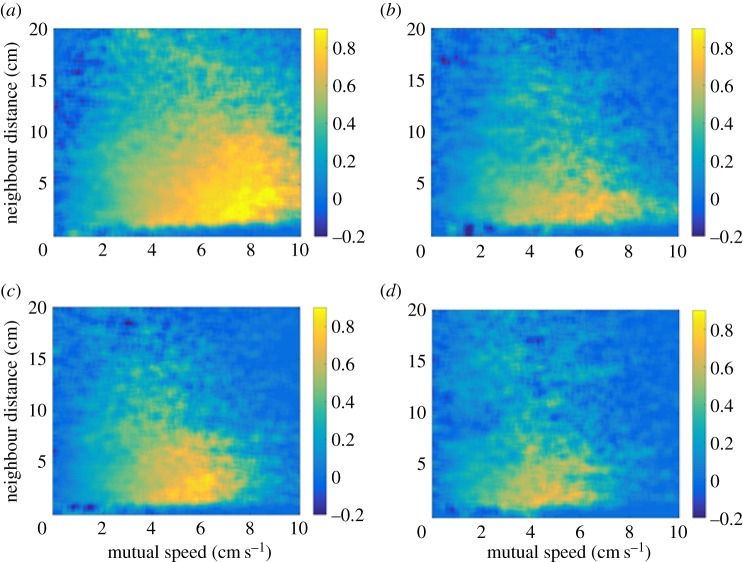
Heat maps depicting the directional correlation, *r*, of pairs of fish as a function of the distance between the pair and the mutual speed of the pair. Panel (*a*) shows familiar fish at interval 1; (*b*) shows unfamiliar fish at interval 1; (*c*) shows familiar fish at interval 2 and (*d*) shows unfamiliar fish at interval 2.

**Figure 3.**
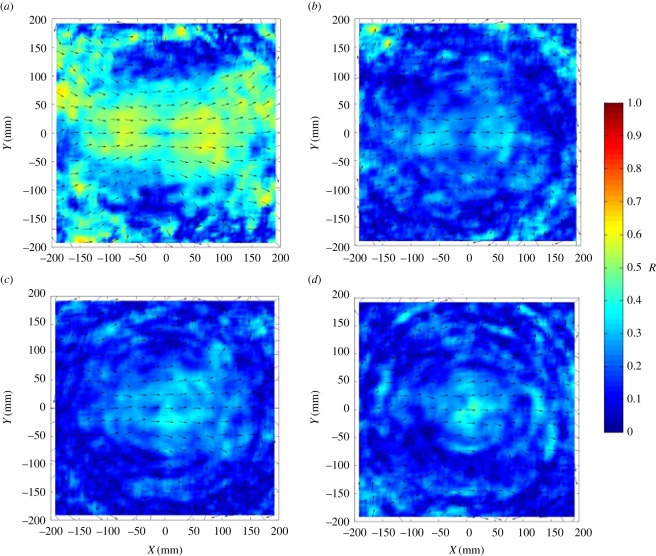
The alignment of group members, relative to a focal individual positioned at the origin and travelling parallel to the positive *x*-axis. Panels (*a,b*) show the familiar treatment at the first and second sampling interval, respectively. Panels (*c,d*) show the unfamiliar treatment at the first and second sampling intervals, respectively. Arrows represent the mean direction of motion of partner fish at different spatial locations. The surface heat plots represent the polarization of the set of differences in angle between focal individuals at the origin and their partners at given relative (*x*, *y*) coordinates, *R*. (See the electronic supplementary material for more details on the generation of these plots.)

The average directional correlations between pairs of fish with respect to their mutual speed and the distance between them are depicted in figure 2. The correlations are generally greater (more positive) as mutual speed increases, and when the distance between the pair is small. Fish in familiar groups appear to show greater directional correlations, especially in the first sampling period. Similarly, figure 3 shows greater alignment of fish in familiar groups during the first sampling period, particularly in the vicinity of the focal fish.

## 4. Discussion

Shoals comprising familiar fish show significant differences in their patterns of collective motion in comparison to shoals of unfamiliar fish. Most strikingly, polarization is greater in familiar shoals. Allied to this result, the movements of fish in familiar shoals were more strongly correlated than those in unfamiliar groups, particularly during the first sampling time interval. Similarly, the alignment of fish was greater for a wide range of shoal-mate positions in familiar shoals, again, particularly during the first sampling period. Taken together, these results show that fish in familiar shoals show greater coordination and more coherent behaviour across the group than unfamiliar fish, particularly when the environment is novel. Polarization in the familiar groups decreased as the fish habituated to the novel conditions, so that there was little obvious difference between the treatments during the second sampling period. This supports the idea that polarization may be an adaptive response to the perception of risk in prey species, and that groups comprising familiar individuals may be better able to cope with such challenges [25].

Surprisingly, there was only a weak trend towards greater cohesion in familiar groups, in contrast to the results of Chivers *et al*. [13]. This may be the result of the fish clustering in response to their introduction to a novel environment during the first sampling period. Mean nearest-neighbour distance appears greater in the second sampling period in the unfamiliar groups, while nearest-neighbour distances remained similar across both periods in the familiar treatment. Regardless of the similarity in mean nearest-neighbour distances between familiar and unfamiliar groups during the first sampling period, there are considerable differences in the alignment of fish between the treatments at this time. This suggests that cohesion on its own does not drive the alignment of group members.

Preferences for affiliating with familiars, and stronger social connections that link such individuals, probably affect both the structure and function of groups and the coherence of collectively moving groups [26–28]. If individuals tend to align more strongly with familiar than with unfamiliar conspecifics, this may produce patterns of localized alignment in sub-groups within larger animal aggregations. Ultimately, it may result in group fission and the passive assortment of animals into familiar groups.

Studies of social interactions have suggested that groups comprising unfamiliar individuals do not function optimally, because individuals divert their attention to the cognitively demanding task of assessing strangers in their social environment [29–31]. Building on this, our study suggests that social familiarity can promote the tendency and the ability of animals to align with one another during collective motion. Ultimately, this may serve to produce more ordered groups and which may strengthen communication networks. It would be valuable in the future to examine whether this is the case and to test whether this results in information being transferred more effectively between familiar individuals.

## Supplementary Material

Supplementary Information: Data and Calculations Used

## Data accessibility

The data are available in the electronic supplementary material.
