# Supplementary material for: Familiarity affects collective motion in shoals of guppies (Poecilia reticulata)
Source: R Soc Open Sci. 2017 Sep 20;4(9):170312. doi: 10.1098/rsos.170312 (PMC5627077; doi:10.1098/rsos.170312)
Supplement: Supplementary Information: Data and Calculations Used [file rsos170312supp1.docx]

**Familiarity affects collective motion in shoals of guppies (*Poecilia reticulata*) –**

**Supplementary Information**

Scarlet Davis, Ryan Lukeman, Timothy M Schaerf and Ashley J W Ward

*Visualisation of alignment of group members relative to focal fish*

We determined the mean relative alignment in the directions of motion of guppies with their groupmates, as a function of the relative locations of groupmates, and rendered the result using MATLAB’s built-in *quiver* function. In addition, we determined the polarisation of angular differences that occurred between focal guppies and their group mates when group mates occupied particular relative locations. Our overall visualisation methods were inspired by the work of (1), which was then built upon in (2-6).

For each guppy, we performed a series of preliminary calculations to estimate the fish’s velocity, speed and from these, a component-wise breakdown of the fish’s direction of motion. Writing $\left(x_{i}(t), y_{i}(t)\right)$ as the coordinates of guppy *i* at time *t* (as determined by IDTracker), we first determined the *x* and *y* components of each guppy’s velocity using the standard forward-difference approximations:

$u_{i}(t) = \frac{x_{i}\left(t + \Delta t\right) - x_{i}\left(t\right)}{\Delta t} \text{and} v_{i}(t) = \frac{y_{i}\left(t + \Delta t\right) - y_{i}\left(t\right)}{\Delta t},$ (1)

where $\Delta t = 1/25$s was the constant duration between consecutive video frames. We then estimated each guppy’s speed (magnitude of velocity) at time *t* via:

$s_{i}(t) = \sqrt{\left(u_{i}(t)\right)^{2} + \left(v_{i}(t)\right)^{2}}$. (2)

Using equations (1) and (2), we constructed unit vectors in the direction of motion of each guppy *i* for each time *t* with components

(3)

in the *x* and *y* directions respectively.

The overarching goal of the next sequence of calculations was then to determine relative coordinates of each fish *j*, relative to both the current location and direction of motion of fish *i*.

We determined the distance between every pair of guppies, *i* and *j*, for all times *t*, using the standard distance formula:

$d_{i, j}(t) = \sqrt{\left(x_{j}(t) - x_{i}(t)\right)^{2} + \left(y_{j}(t) - y_{i}(t)\right)^{2}}$. (4)

Additionally, we determined the internal angle between the directions of motion of each pair of fish, taking into account if the direction of motion of fish *j* was anti-clockwise or clockwise relative to the direction of motion of fish *i*. To do this, we first calculated the internal angle between the directions of motion of each fish using:

, (5)

(the inverse cosine of the dot product of the unit vectors in the directions of motion of fish *i* and *j*). Equation (5) returns an angle such that $0 \leq\alpha_{i, j}(t) \leq\pi$ (radians) (or $0^{\circ} \leq\alpha_{i, j}(t) \leq180^{\circ}$); to determine if the direction of motion of fish *j* was clockwise or anticlockwise relative to the direction of motion of fish *i* within the bounds of the output of equation (5) we performed an additional calculation. We examined the sign of the vertical component of the cross product of the unit vectors in the directions of motion of fish *i* and fish *j*:

. (6)

Taking into account the clockwise/anti-clockwise rotation from the direction of motion of guppy *i* to the direction of motion of guppy *j*, the angle between the directions of motion of the pair of fish was then given by:

$\vartheta_{i, j}(t) = \left\{\begin{matrix} \psi_{i, j}(t)\alpha_{i, j}(t) & if \psi_{i, j}(t) \neq0, \\ \alpha_{i, j}(t) & if \psi_{i, j}(t) = 0. \end{matrix}\right.$ (7)

Next, we calculated the angle between the direction of motion of each guppy *i* (which we identified as a *focal* individual in this context) and the directed straight line segment from the location of guppy *i* to all other group mates *j* (*partner* fish in this context) in turn, for all time steps/video frames *t*. The calculations that follow were repeated treating all fish *i* in turn as focal fish, and all other group mates as partners (for *j ≠ i*). We determined the unit vector in the direction of the straight line segment from each fish *i* to each partner *j*, with components:

. (8)

The internal angle between the unit vectors representing the direction of motion of fish *i* and the direction from fish *i* to fish *j* was then determined via:

(9)

(the inverse cosine of the dot product of the unit vectors in the direction of motion of fish *i* and the direction from fish *i* to fish *j*). Analogous to the determination of the angle between the directions of motion of two fish, we examined the sign of the vertical component of the cross product of the unit vector in the direction of motion of fish *i* with the unit vector pointing from fish *i* to fish *j* to determine if fish *j* was positioned to the left or right of fish *i*:

. (10)

Combining the information from equations (9) and (10), the signed angle between the direction of motion of fish *i* and the line to the location of fish *j* was:

$\phi_{ij}(t) = \left\{\begin{matrix} \lambda_{ij}(t)\varphi_{ij}(t) & \text{if} \lambda_{ij}(t) \neq0, \\ \varphi_{ij}(t) & \text{if} \lambda_{ij}(t) = 0. \end{matrix}\right.$ (11)

The ultimate result of these calculations was the polar coordinates of fish *j* relative to fish *i*'s position and direction of motion $\left(d_{i, j}(t), \phi_{ij}(t)\right)$. For subsequent calculations, we converted these polar coordinates to Cartesian coordinates via:

$$\begin{matrix} x_{ij, \text{relative}}(t) & = & d_{i, j}(t) \cos\left(\phi_{ij}(t)\right), \\ y_{ij, \text{relative}}(t) & = & d_{i, j}(t) \sin\left(\phi_{ij}(t)\right). \end{matrix}$$

We divided the domain where $-200 < x_{ij, \text{relative}}(t) \leq200$, $-200 < y_{ij, \text{relative}}(t) \leq200$ (in millimetres), centred on each focal fish *i*, into a set of overlapping square bins of side length 16 mm, such that the left edges and bottom edges of consecutive bins were separated by 4 mm. In other words the left edges of the bins were located at $x_{l, \text{left}} = -200, -196, \ldots, 184$ (mm), the right edges of the bins were located at $x_{l, \text{right}} = -184, -180,\ldots, 200$(mm), the bottom edges of the bins were located at $y_{k, \text{bottom}} = -200, -196, \ldots, 184$ (mm) and the top edges of the bins were located at $y_{k, \text{top}} = -184, -180,\ldots, 200$ (mm).

For all fish *i*, for every time step (looping over all partner fish *j ≠ i* and across all data from the same treatment and time interval), we included the angular difference between the directions of motion of fish *i* and *j*, as given by equation (7), in bin $\left(l, k\right)$ if $x_{l, \text{left}} < x_{ij, \text{relative}}(t) \leq x_{l, \text{right}}$ and $y_{k, \text{bottom}} < y_{ij, \text{relative}}(t) \leq y_{k, \text{top}}$. Once data corresponding to all fish and time steps was allocated to bins, we determined the mean and polarisation of the set of angular differences contained in each bin using standard methods of circular statistics (see for example (7)). For reference, the mean, $\bar{\vartheta}$, of a set of angles in a given bin, $\vartheta_{i}$, was determined by:

$\bar{\vartheta} = \tan^{-1}\left(\frac{Y}{X}\right)$, (12)

where $X = \sum_{i = 1}^{n} \cos\vartheta_{i}, Y = \sum_{i = 1}^{n} \sin\vartheta_{i}$, $n$ was the number of elements in the bin and care was taken to ensure that $\bar{\vartheta}$ was in the correct quadrant based on the signs of $X$ and $Y$(in practice we used MATLAB’s intrinsic *atan2* function to ensure the correct value for $\bar{\vartheta}$). The polarisation of the angular differences contained in a given bin was calculated via:

$R = \frac{\sqrt{X^{2} + Y^{2}}}{n}$. (13)

We rendered the values of $R$across the set of bins using MATLAB’s *surf* function, with each value of $R$ associated with the coordinates at the centre of each bin. We then superimposed arrows indicating the mean direction of motion of partner fish at different spatial locations relative to focal fish (located at the origin, and moving parallel to the positive *x*-direction by virtue of the calculations performed to determine $x_{ij\text{,} \text{relative}}$ and $y_{ij\text{,} \text{relative}}$) using MATLAB’s *quiver* function. To avoid cluttering of our image, we only plotted arrows indicating the mean direction of motion of partner fish in every sixth bin in the *x*- and *y*- directions.

*Directional Correlation*

For the directional correlation of fish i and j, we computed the unit vector in the direction of each fish, *u_i_* and *u_j_*, (*u_i_* = *v_i_*/*s_i_*, where *v_i_* is the velocity vector and *s_i_* is the speed) and defined the correlation *d*(*i,j*) as the dot product of the two unit vectors, *d*(*i,j*) = *u_i_* · *u_j_*. Defined in this way, perfectly aligned fish have a correlation of 1, fish heading opposite have a correlation of -1, and a randomly disordered group will have an average correlation tending to 0. Trajectories were smoothed using a 7 point Savitzky-Golay filter.

*Polarisation*

Polarisation is a standard measure for the degree of instantaneous alignment in facing or movement direction amongst members of a moving group (see for example (8)). Compactly, the polarisation in movement directions in a group of *n* individuals at time *t* can be calculated via:

$$R_{movement}(t) = \frac{\sqrt{\left(U(t)\right)^{2} + \left(V(t)\right)^{2}}}{n}$$

where , , and $\hat{u}_{i}(t)$ and $\hat{v}_{i}(t)$ are the components of a unit vector pointing in the direction of motion of individual *i* at time *t* (as given by equation (3) in the section “*Visualisation of alignment of group members relative to focal fish*” above). Geometrically, $R_{movement}(t)$ is the length of the resultant vector obtained by adding together the unit vectors in the direction of motion of each individual at time *t*, divided by the total number of group members. If the group members are all travelling in exactly the same direction at a given time, then $R_{movement}(t) = 1.$Values of $R_{movement}(t)$ close to 1 indicate very close agreement in directions of motion, and in such a case the group could be referred to as “highly polarised”. At the other end of the scale, values of $R_{movement}(t)$ close to 0 suggest a high degree of dispersion in directions of motion across group members.

**Data**

Group Data

| Group | Treatment | Time Interval | Mean Polarisation |
| --- | --- | --- | --- |
| 1 | Unfamiliar | 1 | 0.54877471 |
| 2 | Unfamiliar | 1 | 0.481295182 |
| 3 | Unfamiliar | 1 | 0.537698943 |
| 4 | Unfamiliar | 1 | 0.490274413 |
| 5 | Unfamiliar | 1 | 0.562595913 |
| 6 | Unfamiliar | 1 | 0.60037427 |
| 1 | Unfamiliar | 2 | 0.55649653 |
| 2 | Unfamiliar | 2 | 0.496630452 |
| 3 | Unfamiliar | 2 | 0.43102554 |
| 4 | Unfamiliar | 2 | 0.491077899 |
| 5 | Unfamiliar | 2 | 0.42644 |
| 6 | Unfamiliar | 2 | 0.450437331 |
| 7 | Familiar | 1 | 0.706921416 |
| 8 | Familiar | 1 | 0.623307593 |
| 9 | Familiar | 1 | 0.587069821 |
| 10 | Familiar | 1 | 0.721205632 |
| 11 | Familiar | 1 | 0.611430004 |
| 12 | Familiar | 1 | 0.555483353 |
| 7 | Familiar | 2 | 0.535672747 |
| 8 | Familiar | 2 | 0.452516576 |
| 9 | Familiar | 2 | 0.499339211 |
| 10 | Familiar | 2 | 0.48932506 |
| 11 | Familiar | 2 | 0.486100001 |
| 12 | Familiar | 2 | 0.528363394 |

Individual Data

| Group | ID | Treatment | Time | Median Speed | Mean Nearest Neighbour Distance |
| --- | --- | --- | --- | --- | --- |
| 1 | 1 | Familiar | 1 | 30.60417659 | 56.31810755 |
| 1 | 2 | Familiar | 1 | 32.61504637 | 43.8161912 |
| 1 | 3 | Familiar | 1 | 29.39542907 | 43.33290265 |
| 1 | 4 | Familiar | 1 | 26.47008925 | 37.0328918 |
| 2 | 5 | Familiar | 1 | 29.00565678 | 45.46001221 |
| 2 | 6 | Familiar | 1 | 36.35204602 | 66.79124652 |
| 2 | 7 | Familiar | 1 | 29.76613008 | 65.92796367 |
| 2 | 8 | Familiar | 1 | 13.91673902 | 105.4844402 |
| 3 | 9 | Familiar | 1 | 30.28997359 | 52.53058498 |
| 3 | 10 | Familiar | 1 | 31.09446293 | 77.56662545 |
| 3 | 11 | Familiar | 1 | 22.28857611 | 112.066574 |
| 3 | 12 | Familiar | 1 | 26.62818056 | 62.27562141 |
| 4 | 13 | Familiar | 1 | 32.93232644 | 38.22625781 |
| 4 | 14 | Familiar | 1 | 36.85410425 | 41.13928104 |
| 4 | 15 | Familiar | 1 | 33.06067339 | 49.081465 |
| 4 | 16 | Familiar | 1 | 32.56631695 | 43.55114961 |
| 5 | 17 | Familiar | 1 | 21.21068422 | 35.65647128 |
| 5 | 18 | Familiar | 1 | 24.90230913 | 49.72768043 |
| 5 | 19 | Familiar | 1 | 23.14088644 | 56.50425201 |
| 5 | 20 | Familiar | 1 | 20.66712365 | 37.57415647 |
| 6 | 21 | Familiar | 1 | 16.14088365 | 46.70057224 |
| 6 | 22 | Familiar | 1 | 18.17706316 | 31.69463534 |
| 6 | 23 | Familiar | 1 | 18.79680824 | 26.76667165 |
| 6 | 24 | Familiar | 1 | 14.19650045 | 39.93812865 |
| 1 | 1 | Familiar | 2 | 17.56989613 | 41.47047924 |
| 1 | 2 | Familiar | 2 | 10.67906831 | 104.8373034 |
| 1 | 3 | Familiar | 2 | 19.68700396 | 67.68551392 |
| 1 | 4 | Familiar | 2 | 16.32490429 | 33.04615811 |
| 2 | 5 | Familiar | 2 | 3.74040439 | 88.59913098 |
| 2 | 6 | Familiar | 2 | 10.45361779 | 65.52367203 |
| 2 | 7 | Familiar | 2 | 8.087992643 | 48.919557 |
| 2 | 8 | Familiar | 2 | 18.28284715 | 67.5665514 |
| 3 | 9 | Familiar | 2 | 11.60226271 | 119.6515543 |
| 3 | 10 | Familiar | 2 | 13.91673902 | 107.5976944 |
| 3 | 11 | Familiar | 2 | 18.94109289 | 71.11267596 |
| 3 | 12 | Familiar | 2 | 15.0698988 | 90.04328473 |
| 4 | 13 | Familiar | 2 | 19.40845499 | 107.3558013 |
| 4 | 14 | Familiar | 2 | 13.04439535 | 28.74470858 |
| 4 | 15 | Familiar | 2 | 10.35549371 | 72.58442779 |
| 4 | 16 | Familiar | 2 | 13.54217948 | 23.63612448 |
| 5 | 17 | Familiar | 2 | 8.855683203 | 37.6049999 |
| 5 | 18 | Familiar | 2 | 8.254582061 | 33.46564018 |
| 5 | 19 | Familiar | 2 | 10.69675769 | 45.06886583 |
| 5 | 20 | Familiar | 2 | 11.87605258 | 36.05510329 |
| 6 | 21 | Familiar | 2 | 13.7609956 | 31.60121864 |
| 6 | 22 | Familiar | 2 | 11.8090008 | 30.29097504 |
| 6 | 23 | Familiar | 2 | 16.09396238 | 38.83051749 |
| 6 | 24 | Familiar | 2 | 17.15832524 | 26.77864431 |
| 7 | 25 | Unfamiliar | 1 | 21.22894913 | 45.39429898 |
| 7 | 26 | Unfamiliar | 1 | 31.23230099 | 68.22684343 |
| 7 | 27 | Unfamiliar | 1 | 34.71376969 | 42.60704949 |
| 7 | 28 | Unfamiliar | 1 | 14.8348418 | 103.6658216 |
| 8 | 29 | Unfamiliar | 1 | 7.511075156 | 72.50117101 |
| 8 | 30 | Unfamiliar | 1 | 17.83264212 | 62.51980294 |
| 8 | 31 | Unfamiliar | 1 | 16.96328756 | 106.2490619 |
| 8 | 32 | Unfamiliar | 1 | 14.51260228 | 88.28615768 |
| 9 | 33 | Unfamiliar | 1 | 19.15153388 | 68.70509954 |
| 9 | 34 | Unfamiliar | 1 | 12.31373725 | 47.06330823 |
| 9 | 35 | Unfamiliar | 1 | 11.55 | 49.58695955 |
| 9 | 36 | Unfamiliar | 1 | 13.58399794 | 53.11502695 |
| 10 | 37 | Unfamiliar | 1 | 16.11509386 | 74.33508608 |
| 10 | 38 | Unfamiliar | 1 | 18.01617329 | 56.11860272 |
| 10 | 39 | Unfamiliar | 1 | 20.64515682 | 69.32825516 |
| 10 | 40 | Unfamiliar | 1 | 14.389623 | 87.84381286 |
| 11 | 41 | Unfamiliar | 1 | 17.77423518 | 47.14250738 |
| 11 | 42 | Unfamiliar | 1 | 15.74960317 | 76.97773908 |
| 11 | 43 | Unfamiliar | 1 | 22.0635021 | 44.39736133 |
| 11 | 44 | Unfamiliar | 1 | 25.33584417 | 47.88852933 |
| 12 | 45 | Unfamiliar | 1 | 8.47160699 | 96.60774454 |
| 12 | 46 | Unfamiliar | 1 | 24.62747449 | 118.9156948 |
| 12 | 47 | Unfamiliar | 1 | 21.47818661 | 50.14032727 |
| 12 | 48 | Unfamiliar | 1 | 20.62683325 | 74.7785044 |
| 7 | 25 | Unfamiliar | 2 | 21.84083518 | 51.53402361 |
| 7 | 26 | Unfamiliar | 2 | 29.00531783 | 93.44229938 |
| 7 | 27 | Unfamiliar | 2 | 27.19473318 | 107.4285493 |
| 7 | 28 | Unfamiliar | 2 | 25.66797616 | 59.80769961 |
| 8 | 29 | Unfamiliar | 2 | 11.60552132 | 118.2863209 |
| 8 | 30 | Unfamiliar | 2 | 13.90586657 | 91.73797284 |
| 8 | 31 | Unfamiliar | 2 | 10.20468642 | 114.2411552 |
| 8 | 32 | Unfamiliar | 2 | 19.22051313 | 75.00808087 |
| 9 | 33 | Unfamiliar | 2 | 5.781543911 | 162.0391197 |
| 9 | 34 | Unfamiliar | 2 | 4.134156504 | 180.4010522 |
| 9 | 35 | Unfamiliar | 2 | 15.10248324 | 178.5693862 |
| 9 | 36 | Unfamiliar | 2 | 5.137423965 | 219.6922984 |
| 10 | 37 | Unfamiliar | 2 | 9.530247898 | 130.3371508 |
| 10 | 38 | Unfamiliar | 2 | 10.85118081 | 78.21845693 |
| 10 | 39 | Unfamiliar | 2 | 12.25216818 | 49.47748739 |
| 10 | 40 | Unfamiliar | 2 | 14.91352155 | 36.9908242 |
| 11 | 41 | Unfamiliar | 2 | 9.231972048 | 70.00288219 |
| 11 | 42 | Unfamiliar | 2 | 10.11162697 | 31.39830214 |
| 11 | 43 | Unfamiliar | 2 | 10.7390933 | 27.21803694 |
| 11 | 44 | Unfamiliar | 2 | 9.462557794 | 31.38756593 |
| 12 | 45 | Unfamiliar | 2 | 12.67687363 | 48.56125034 |
| 12 | 46 | Unfamiliar | 2 | 6.6 | 168.7408003 |
| 12 | 47 | Unfamiliar | 2 | 8.323010273 | 81.1812089 |
| 12 | 48 | Unfamiliar | 2 | 0.275 | 170.603751 |

**References**

1. Lukeman R, Li YX, Edelstein-Keshet L. Inferring individual rules from collective behavior. Proceedings of the National Academy of Sciences of the United States of America. 2010;107(28):12576-80.

2. Katz Y, Tunstrom K, Ioannou CC, Huepe C, Couzin ID. Inferring the structure and dynamics of interactions in schooling fish. Proceedings of the National Academy of Sciences of the United States of America. 2011;108(46):18720-5.

3. Herbert-Read JE, Perna A, Mann RP, Schaerf TM, Sumpter DJT, Ward AJW. Inferring the rules of interaction of shoaling fish. Proceedings of the National Academy of Sciences of the United States of America. 2011;108:18726-31.

4. Schaerf TM, Herbert-Read JE, Myerscough MR, Sumpter DJT, Ward AJW. Identifying differences in the rules of interaction between individuals in moving animal groups. arXiv preprint. 2016;arXiv:1601.08202.

5. Schaerf TM, Dillingham PW, Ward AJW. The effects of external cues on individual and collective behaviour of shoaling fish. Science Advances. 2017;3(6):e1603201. DOI: 10.1126/sciadv.1603201

6. Ward AJW, Schaerf TM, Herbert-Read JE, Morrell LJ, Sumpter DJT, Webster MM. Local interactions and global properties of free-ranging stickleback shoals. Royal Society Open Science. 2017; 4(7): 170043.

7. Zar JH. Biostatistical Analysis. Upper Saddle River, NJ: Prentice Hall; 1996.

8. Couzin ID, Krause J, James R, Ruxton GD, Franks NR. Collective memory and spatial sorting in animal groups. Journal of Theoretical Biology. 2002;218(1):1-11.
